# Miscellaneous Intelligence

**Published:** 1801-02

**Authors:** 


### MISCELLANEOUS INTELLIGENCE.

CIT. VAQUELIN has diſcovered in *the common houſe leek (ſempervivum tectorum L.)* a great quantity of malat of lime (malate de chaux) even in a greater proportion than it is met with in any fruit. It may be extracted in two different ways; the one by evaporating the juice, and pouring alkohol on the reſiduum to free it from the ſaccharine parts. After being ſufficiently waſhed, it is treated with half its weight of ſulphuric acid, mixed with ſeven or eight parts of water But as by this method, a portion of ſulphat of lime is left in the malic acid, the following ſeems to be preferable. A ſolution of acetite of lead is poured upon the juice of the ſempervivum; and after having edulcorated the precipitate, it is decompoſed by diluted ſulphuric acid. In the ſame way the pureſt malic acid acid may be obtained, which is but very little impregnated with colouring particles. The fame plant contains likewife a confiderable portion of faccharine matter, and affords alfo a fort of brandy: And after having feparated the malat of lime, a fyrup may be got by evaporation, much refembling that of apples. The root gives alfo a great portion of malat of lime. This is the only plant in which it has been found. Hitherto it has been only extracted from fome fruits, nuts, berries, &c. It is very probable that malic acid is contained in the fame ftate in all fucculent plants, and particularly in the genus of *Sedum*. (*Annales de Chemie*, 101.)

---

According to the refearches of the fame chemift, *the vitrum antimonii*, or glafs of antimony, contains in 100 parts, 12 parts, or more frequently 9 or 10 parts of *filiceous earth*. It is probable, that it originates from the crucibles in which the oxyd of antimony is melted on the filiceous earth, on which this oxyd has, like that of lead, a great action. It is eafily conceived, that its proportion muft vary according to the degree of heat that is employed, and the time it is kept melting. It is likewife poffible, that this earth comes from the furnace in which the fulphur of antimony is calcined. This circumftance, undoubtedly, accounts for the inconftant effects of emetic tartar, which is always produced, if the filiceous earth is not feparated by repeated cryftallizations, or if the folution is merely evaporated, a practice ftill very common amongft apothecaries. Many perfons will probably wonder how filiceous earth fhould diffolve in tartaric acid, as it refifts the action of acids much ftronger than this; but it may be remarked, that as foon as it is united with any alkali, earth, or oxyd of metal, a weak acid is capable of diffolving it.

It is therefore advifable in preparing emetic tartar, to diffolve as much of the vitrum antimonii in cremor tartari as is poffible, to filter the folution warm, and let it afterwards evaporate till the refiduum becomes dry. This is again diffolved in hot water, and left to cryftallize. By this means all filiceous earth diffolved in the acid feparates at the end of the evaporation, and diffolves no more; and at the fame time the emetic is obtained in a greater quantity and a purer ftate, than when the earth oppofes the cryftallization. (*Ibid.*)

---

Mr. Hermbstaedt mentions in a letter to Profeffor Scherer of Halle, that he is preparing a new edition of his Experimental Chemiftry, in which he confiders the calorique or calorific principle, not as warm itfelf, but only as producing heat and the fenfation of warmth merely as a phenomenon of extenfion, which affects our nerves, as warmth; on this account he calls the calorique, *thermogenium*. He likewife confiders the light as lighting or illuminating, when it affects our eyes, and names it light-producing principle, *Photogenium*. To the unknown acidifiable bafis of Borax, he affigns the name of *Boraceum*; that of the fluoric acid he diftinguifhes with the name of *Fluoreum*; that of muriatic acid with

*Muriacum*,

*Muriacum.* He differs from Richter by confidering the light-producing principle as fimple and not yet decompofed; but he allows, at the fame time, that it is contained in all incombuftible fubftances. (*Scherer's Journal of Chemiftry*, in German, *Vol. IV. No.* 22, 1800.)

---

We are informed, that Mr. Blair, of the Lock Hofpital and Finfbury Difpenfary, affifted by feveral other refpectable furgeons, has been fome time engaged in writing a comprehenfive *Syftem of Medical and Operative Surgery*, adapted to the prefent improved practice at the London Hofpitals, &c. We fhall take an early opportunity of laying before our readers a more particular account of this undertaking, as a Profpectus is faid to be in the prefs.

---

Mr. William Henry, of Manchefter, has in the prefs, and in confiderable forwardnefs, a fmall work, intended, partly, to facilitate the acquirement of chemical knowledge to perfons entering on this ftudy without the benefit of an inftructor; and, partly, as a portable companion for the ufe of more advanced ftudents. The firft part will contain directions refpecting the mode of learning chemiftry, and, alfo, an arranged feries of experiments, neceffary to be performed by thofe who intend to become acquainted, by actual obfervation, with the chemical properties and habitudes of bodies. More minute and precife directions will be given for conducting thefe experiments with fuccefs, than are to be found in other elementary books. The fecond part will comprife, Summary Inftructions for the Analyfis of Mineral Waters and of Mineral Bodies in general. And the third part will point out fome of the ufeful applications of chemical agents in detecting adulterations, difcovering poifons, &c. The work will form one fmall pocket volume; and it may be proper to remark, that it will not at all interfere with the excellent little Manual lately publifhed by Mr. Parkinfon, the plan and objects of which are perfectly different.

---

Dr. Ferguson, of Aberdeen, has in the prefs an Effay on Fever, which will be publifhed in a few months.

---

Dr. Pearson's Lectures on the Practice of Phyfic, Materia Medica, and Chemiftry, will re-commence in the Elaboratory, Whitcomb Street, Leicefter Square, on Tuefday, February the 3d, at eight o'clock in the morning.

